# Chemistry and Bacteriology

**Published:** 1890-08

**Authors:** John A. Miller

**Affiliations:** Buffalo, N. Y., Professor of Chemistry, Niagara University


### CHEMISTRY AND BACTERIOLOGY.

BY JOHN A. MILLER, PH. D., Buffalo, N. Y.,
Professor of Chemistry, Niagara University.

### RECENT RESEARCHES ON PTOMAINES.

*Immunity against septicemia induced by soluble substances.* By Roux and Chamberland, (Annal. de l'institute Pasteur, 1888. *Jour. Analy. Chem. IV.*, 108).

Cultures of the bacillus of malignant edema were sterilized and filtered, and portions of the fililate injected into the abdominal cavity of guinea-pigs every third day. Some disturbances were produced, but subsequent injections were borne without apparent effect. Two days after the last injections, these animals, together with others used as controls, were inoculated with virulent germs of malignant edema. The control animals all died within eighteen hours, while those which had received the sterilized cultures remained permanently unaffected.

A second series of experiments seemed to show that the heating of the cultures to 105° – 110° partially decomposes the chemical poisons. The serose fluids from the tissues of guinea-pigs dead from malignant edema were rendered germ-free; 40 cc. killed healthy animals in a few hours with all the characteristic symptoms of malignant edema, while the same quantity of the heated culture produced no effect. Seven or eight inoculations of 1 cc. of this fluid secured immunity against subsequent inoculations with the germs.

The experiments also show that in certain media the cultures of the germs do not produce substances which induce immunity.

*Immunity against charbon symptomatique induced by soluble substances.*— (Roux, ibid; ibid.) Experiments similar to the above show that the bacillus of charbon symptomatique, as well as that of malignant edema, produces a chemical poison, and that by the establishment of a tolerance for the poison immunity against the germs can be secured for a time.

*The value of iodoform in the dressing of suppurating wounds.*—Behring (*Deut. Med. Wochenschrift*, 1889, 653; ibid) claims that the value of iodoform is not due to its germicidal properties, but to the fact that it renders chemically inert the ptomaine, cadaverine. This position is supported by De Ruyter and by Warle.

*Ptomaine of Hydrophobia.*—Aurep (Proc. Med. Soc. of St. Petersburg, 1889; ibid,) obtained by Brieger's method from the brains of rabbits, in which hydrophobia had been induced, an alkaloidal substance of highly poisonous properties. Chemical composition not determined.

Small doses (1-100 to 3-100 mgrms.) produced in rabbits an elevation of temperature, restlessness, rapid breathing, and palpitation of the heart; medium doses (5-100 to 2-10 mgrms.) difficult respiration, tremor, weakness of the heart, and general loss of strength. Large doses (3-10 to 5-10 mgrms.) are followed by depression of temperature, paralysis of the extremities, salivation, and death, with symptoms of asphyxia. In these symptoms it is claimed that we have the complete chemical history of hydrophobia, the effects of the smaller doses corresponding with first, and those of the larger doses with later stage of the disease.

